# Inflammation and Immune Mechanisms in Atherosclerosis

**DOI:** 10.31083/RCM49118

**Published:** 2026-08-13

**Authors:** Zihao Wan, Xu Xu, Guofu Zhu

**Affiliations:** ^1^Cardiology Department, The Second Affiliated Hospital of Kunming Medical University, 650101 Kunming, Yunnan, China; ^2^General Medicine Department, Affiliated Calmette Hospital of Kunming Medical University, 650000 Kunming, Yunnan, China

**Keywords:** atherosclerosis, cardiovascular disease, immunity, inflammation

## Abstract

Cardiovascular diseases (CVDs) are the leading cause of death and disability worldwide, and atherosclerosis (AS) is a major underlying pathology. This review systematically examines the interplay between inflammation and immunity in AS. Disease initiation involves endothelial injury, formation of oxidized low-density lipoprotein (ox-LDL), and innate immune responses, including monocyte and macrophage infiltration and dendritic cell (DC) activation. Macrophages polarize to a proinflammatory M1 phenotype, phagocytose lipids to form foam cells, and release inflammatory mediators such as interleukin-1β (IL-1β) and tumor necrosis factor-α (TNF-α), thereby exacerbating plaque inflammation. DCs serve as a crucial link between innate and adaptive immunity by presenting antigens to CD4^+ ^T cells. T helper 1 (Th1) cells facilitate inflammation through interferon-γ (IFN-γ), whereas regulatory T cells (Tregs) exert protective, anti-inflammatory effects. B cells have dual functions: B1 cells secrete immunoglobulin M (IgM) and provide protection, while B2 cells typically contribute to disease progression. In advanced lesions, immune cells cluster within the arterial wall to form arterial tertiary lymphoid organs (ATLOs), and the identification of neuro–immune–cardiovascular interfaces (NICIs) underscores the involvement of the nervous system. Chronic inflammation results in the thinning of the fibrous cap, thereby heightening the risk of plaque rupture and acute clinical events. Current therapies primarily target inflammatory pathways, including statins, colchicine, and proprotein convertase subtilisin/kexin type 9 inhibitors (PCSK9i), whereas future strategies may focus on directly targeting immune cells.

## 1. Introduction

Atherosclerosis (AS), a chronic inflammatory disease of the arterial wall, remains a leading cause of morbidity and mortality worldwide and is increasingly prevalent among younger populations in developing countries [1,2]. Plaque rupture can occur unexpectedly, leading to acute ischemia and consequent functional impairment of organs and tissues [3]. This condition significantly diminishes the quality of life of patients and, in severe cases, can result in death. AS is a multifaceted process involving various cell types and inflammatory factors. Current literature indicates a close association between the inflammatory processes involved in AS and comorbidities such as hypertension, hyperlipidemia, and diabetes [4]. Although the disease is initiated by lipid deposition and endothelial dysfunction [5,6], its sustained progression, particularly the transition from stable plaque to rupture-prone lesions, is driven by a complex interplay between innate and adaptive immunity in the context of chronic inflammation [7]. The roles of immune cells, such as macrophages and T cells, and inflammatory mediators, including IL-1β, TNF-α, and interleukin-6 (IL-6), in promoting endothelial activation, smooth muscle cell phenotype switching, and plaque destabilization are well established [8,9,10]. However, translating this mechanistic understanding into clinical practice remains challenging and, at times, controversial. For example, systemic inflammatory markers such as high-sensitivity C-reactive protein (hs-CRP) are strongly associated with cardiovascular event risk; however, the precise causal pathways and optimal therapeutic targets within the inflammatory cascade remain under investigation.

The development of AS is influenced by inflammatory and immune factors, as well as specific immune components. Recent studies have categorized the immune response in AS into innate and adaptive immunity [11]. The innate immune system is composed primarily of macrophages and dendritic cells (DCs) [12,13], whereas adaptive immunity predominantly involves T lymphocytes, which, after decades of research, have been shown to be enriched in the fibrous cap of atherosclerotic lesions [8]. Emerging research has also indicated that, in advanced AS, a neuro–atherosclerotic circuit forms in the brain, while peripheral nerve ganglia at the neurovascular interfaces undergo stable remodeling [14]. These nerves transmit signals to the central nervous system, which, through efferent pathways, modulates plaque growth and intensifies inflammatory and immune responses within the plaques [15,16].

Inflammatory factors exert particularly profound effects on plaques, in parallel with immune-related pathological changes. Indeed, some studies have indicated that cytokines, chemokines, and oxidized lipoproteins drive leukocyte activation and contribute to plaque instability [17,18,19]. The release of inflammatory factors typically follows the infiltration of immune cells. These factors, released by immune cells, further modify cellular pathology, triggering a cascade of inflammatory reactions that lead to irreversible disease progression. Common inflammatory factors such as IL-1β, TNF-α, and IL-6 [20] can amplify the inflammatory response of endothelial cells. Given these complexities, this review transcends a general overview of inflammation in AS by: (1) critically summarizing the roles of key immune cell subsets and inflammatory pathways at various stages of the disease; (2) linking mechanistic insights to clinical markers (*e*.*g*., hs-CRP, plaque imaging) and patient outcomes; (3) discussing the translational potential and challenges of emerging therapies targeting the immune–inflammatory axis, with reference to recent clinical trial data. This focused analysis aims to clarify existing controversies and underscore how immunity and inflammation collectively drive the initiation, progression, and clinical outcomes of AS.

## 2. Methodology and Editing Tools

### 2.1 Methods

This study is a narrative review designed to thoroughly examine the inflammatory and immune mechanisms underlying AS. The selection of literature was informed by the expertise of the authors in the field.

A comprehensive search of PubMed, Web of Science, and Google Scholar was performed, focusing on publications from 1995 to 2025. The search terms included “atherosclerosis”, “inflammation”, and “pertinent immune components”. We initially identified 1526 relevant documents in the specified literature database. Ultimately, 125 documents were selected as references, comprising 77 original research articles and 48 review articles. Among the original studies, 22 were clinical investigations. Priority was placed on original research, high-quality reviews, and significant clinical trials that elucidate immune pathways in AS. Studies that focused solely on lipid metabolism, were not peer-reviewed, or exhibited substantial methodological limitations, were excluded from the detailed analysis.

The selected literature was evaluated for methodological quality, followed by a qualitative thematic synthesis to summarize key mechanisms and clinical implications. As a narrative review, this work did not implement a formal systematic search protocol with predefined screening procedures. Consequently, while this review offers an expert-informed perspective, it may not encompass all relevant evidence and does not provide quantitative effect estimates. This transparent description of the methodology clarifies the foundation for the ensuing discussion.

### 2.2 Software and Tools

Manuscript preparation was conducted using WPS Office (version 12.1.0.25225; Zhuhai, GuangDong, China). Reference management was performed with EndNote X9 (Clarivate Analytics, London, UK). For figure preparation, we used Adobe Illustrator 2026 (version 30.0), developed by Adobe Inc. (345 Park Avenue, San Jose, CA, USA), as a professional vector graphics tool to create and edit all diagrams and flowcharts.

## 3. The Involvement of the Immune System in Atherosclerosis

### 3.1 Innate Immunity

#### 3.1.1 Monocytes/Macrophages

AS primarily arises from endothelial cell damage caused by underlying risk factors [5]. This damage, induced by agents such as reactive oxygen species (ROS) and oxidized low-density lipoprotein (ox-LDL) [6], facilitates lipid accumulation and promotes endothelial inflammation. The subsequent infiltration of immune cells, whose composition and proportions dynamically change as the disease progresses, exacerbates lesion development. A pivotal event in AS is the deposition and oxidative modification of lipoproteins, such as ox-LDL, within the arterial intima [21]. These modified lipoproteins activate endothelial cells, vascular smooth muscle cells (VSMCs), and macrophages, thereby initiating and sustaining chronic inflammation [22]. In this inflammatory environment, both macrophages and VSMCs take up lipids and transform into foam cells, with VSMCs contributing a significant proportion of the foam cell population [23]. The eventual apoptosis of these foam cells results in further lipid accumulation and contributes to plaque instability [24]. This process perpetuates the continuous recruitment and infiltration of monocytes. Within the plaque microenvironment, infiltrating macrophages polarize into distinct phenotypes: proinflammatory M1 macrophages, which dominate the necrotic core and amplify inflammation, and anti-inflammatory M2 macrophages, which are less abundant and associated with plaque stabilization [25,26].

The recruitment and infiltration of monocytes and macrophages into AS plaques are primarily driven by classical inflammatory factors and their receptors [27]. For example, in *ApoE^-/-^* mice, downregulation of C–C chemokine receptor 2 (CCR2) and 3 (CCR3) in macrophages attenuates AS progression, highlighting the critical role of immune cell trafficking [19,27]. Additionally, this inflammatory cascade is amplified by adaptive immunity; activated T lymphocytes release interferon-γ (IFN-γ), which promotes macrophage polarization toward the proinflammatory M1 phenotype, thereby facilitating foam cell formation and epigenetic changes [9,10,28]. Other inflammatory mediators, including tumor necrosis factor-α (TNF-α), interleukin-1β (IL-1β), interleukin-6 (IL-6), interleukin-12 (IL-12), and ROS, further activate macrophages, establishing a self-reinforcing inflammatory loop. This loop is initiated and maintained, in part, by innate immune receptors such as the class I scavenger receptor (SREC-I) and Toll-like receptors (TLRs) on macrophages [29].

However, the immune response in AS is complex and not exclusively proinflammatory. Notably, inhibiting macrophage infiltration has been shown to paradoxically shift VSMCs toward a proinflammatory state and increase lymphocyte recruitment, underscoring the delicate balance and potential compensatory mechanisms within the plaque immune microenvironment [19,30,31].

In summary, reducing macrophage infiltration in AS may not effectively halt disease progression, as other immune cells and VSMCs can compensate and continue to drive atherogenesis. Furthermore, macrophage apoptosis within plaques is inevitable, complicating clinical management. Nevertheless, targeting macrophage-derived inflammatory factors or promoting M2 polarization may provide promising therapeutic strategies to slow AS progression [32,33].

#### 3.1.2 Dendritic Cells

While macrophages are the primary cells involved in the innate immune response in AS, neutrophils and DCs also play important roles [34]. Specifically, DCs are crucial as these cells present antigens to other immune cells, thereby bridging the gap between innate and adaptive immunity.

Under the influence of inflammatory factors and the plaque microenvironment, DCs differentiate into various subtypes, including those characterized by CD80, CD83, CD86, and follicular dendritic cells (FDCs) [35,36]. FDCs represent another significant subtype [37]. The primary DC subsets implicated in AS, namely classical dendritic cells (cDCs), plasmacytoid dendritic cells (pDCs), and monocyte-derived DCs, originate from bone marrow progenitors. Upon stimulation with fms-like tyrosine kinase 3 ligand (Flt3L), these progenitors migrate to lymphoid and non-lymphoid tissues, where they undergo further differentiation [38]. Notably, cDCs can develop into either functionally competent or dysfunctional forms [39] and may arise from both bone marrow precursors and macrophages recruited in response to inflammatory signals [19,40,41]. Research indicates that cDCs, particularly the cDC1 subset, promote atherosclerotic inflammation by presenting antigens, activating the *stimulator of interferon genes* (*STING*) pathway, and stimulating CD4^+ ^T cells and cytotoxic CD8^+ ^T cells [39,42]. Meanwhile, pDCs also contribute to inflammation by presenting antigens via major histocompatibility complex class Ⅱ (MHC-II) molecules to activate CD4^+^ T cells, leading to the release of IFN-γ and exacerbation of inflammation. IFN-γ subsequently drives macrophages toward the M1 phenotype through the Janus kinase–signal transducer and activator of transcription (JAK–STAT) pathway, further amplifying the inflammatory response [43,44].

#### 3.1.3 Neutrophils

The prevailing view is that neutrophils are integral components of the innate immune response in AS, and several studies have identified these cells as independent risk factors for disease development [45]. Neutrophils release ROS, which facilitates lipid oxidation and the generation of ox-LDL within plaques [46,47]. Neutrophils also possess the capacity to phagocytose lipids; following this process, neutrophils undergo apoptosis, thereby triggering macrophage phagocytosis and contributing to the expansion of the necrotic core [48]. Furthermore, neutrophils adhere to aortic endothelial cells, thereby promoting lipid deposition and local inflammation. Glucagon-like peptide-1 (GLP-1) receptor agonists, such as semaglutide, can reduce CD11b expression and decrease neutrophil adhesion, ultimately improving disease outcomes [49]. Neutrophils are crucial not only in the early stages but also in the later stages of AS. In advanced plaques, neutrophils secrete matrix metalloproteinases (MMPs), thereby increasing the risk of plaque rupture [50]. Neutrophil extracellular traps (NETs) are also believed to facilitate plaque progression [51,52], and their inflammatory effects interact with those of macrophages. Macrophage-released DNase can degrade NETs, thereby helping to mitigate AS [53]. However, the plaque microenvironment remains highly complex. In late-stage plaques, neutrophil apoptosis and subsequent macrophage activity lead to the release of cytokines and chemokines, thinning the fibrous cap and significantly increasing the risk of plaque rupture and thrombosis [48,54].

### 3.2 Adaptive Immunity

Adaptive immunity, primarily orchestrated by T and B lymphocytes, evolves from the activation of the innate immune system, establishing a bidirectional inflammatory loop within atherosclerotic plaques rather than following a linear pathway [5,55]. This immune response is functionally heterogeneous, with distinct cellular subsets exerting opposing effects on disease progression. A critical unresolved question is how the balance between these pro- and anti-atherogenic forces is dynamically regulated within the human arterial microenvironment, and why therapeutic interventions that are effective in murine models frequently yield conflicting or non-translatable results in humans [56].

#### 3.2.1 The T Cell Spectrum

CD4^+^ T cells serve as crucial regulators that differentiate into functionally diverse subsets. Notably, the aorta can serve as a site of local T cell priming, challenging the conventional view that adaptive immune responses are initiated exclusively in secondary lymphoid organs [8,57].

The proinflammatory axis (Th1/Th17 (T helper 17 cells)): Th1 cells, driven by IFN-γ, are central to the propagation of inflammation, exacerbating endothelial dysfunction and plaque progression [58,59]. However, the role of Th17 cells remains contentious. While these cells are frequently implicated in promoting plaque instability through interleukin-17 (IL-17)-mediated matrix degradation [60,61], evidence also indicates that IL-17 may contribute to fibrous cap stability [62]. This apparent contradiction underscores a significant gap in understanding the contextual signals that dictate the functional fate of Th17 cells in AS.

The regulatory and protective axis (Treg/Th2: regulatory T helper cells, Tregs) is a potent suppressor of inflammation, and the numerical or functional deficiency of Tregs correlates with adverse clinical outcomes [63,64]. A key unresolved issue is the stability of Tregs within the proinflammatory plaque environment, where Tregs may convert into pro-atherogenic Th17-like cells [65]. Th2 cells, typically associated with anti-inflammatory cytokines such as interleukin-4 (IL-4) and interleukin-5 (IL-5), generally mitigate disease [66,67]. However, the mechanisms underlying Th2-mediated protection remain inadequately understood. For instance, IL-5 appears to exert atheroprotective effects independent of its role in antibody production, yet the mechanisms underlying these effects remain unknown [68].

#### 3.2.2 B Lymphocytes

In AS, B cells exhibit a bidirectional role in disease progression. Traditionally, B1 cells are considered to confer protection by secreting natural immunoglobulin M (IgM) [28], whereas B2 cells are associated with disease promotion. However, the role of key regulatory factors such as B-cell activating factor (BAFF) remains contentious. Although BAFF can exacerbate plaque formation by neutralizing IgM [69], systemic inhibition of BAFF may disrupt the transmembrane activator and CAML interactor (TACI) receptor. This disruption can alter the macrophage Toll-like receptor 9–interferon regulatory factor 7 (TLR9–IRF7) pathway, leading to upregulation of C–X–C chemokine ligand 10 (CXCL10) and, consequently, intensifying inflammation [70]. These findings suggest that the BAFF possesses a complex regulatory function that warrants further investigation.

The “dual role” of B2 cells also remains a topic of debate. Their proinflammatory effects are mediated by the activation of Fcγ receptors through immunoglobulin G (IgG)/IgM antibodies [71], while the anti-inflammatory effects attributed to interleukin-10 (IL-10) are also questioned regarding their necessity in the plaque microenvironment [72]. These contradictions suggest that a simplified model based on a limited number of subgroups and molecules, such as IgM and IL-10, may obscure a more intricate regulatory network and functional plasticity within the body. Future research must elucidate the dynamic functions of various B cell subsets across specific disease stages and microenvironments, as well as their interactions with signaling networks. Such complex effects may present potential therapeutic targets.

#### 3.2.3 Synthesis and Translational Outlook

**Fig. 1.**
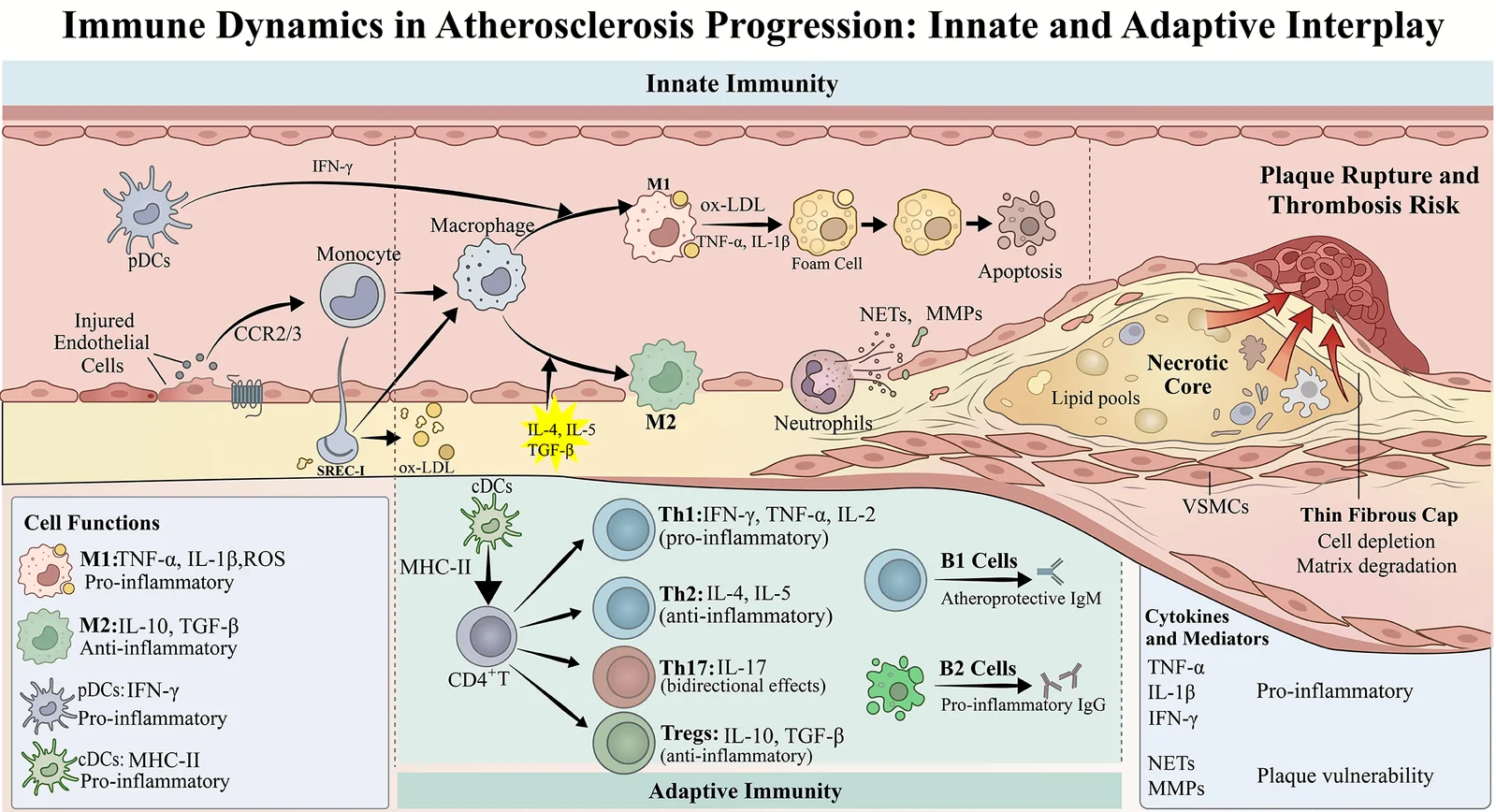
**Roles of innate and adaptive immunity in atherosclerosis (AS)**. Under the influence of initial injurious factors such as oxidized low-density lipoprotein (ox-LDL), hypertension, and diabetes, vascular endothelial cells are damaged and release chemokines, including C–C motif chemokine ligand 2 (CCL2), which initiate the innate immune response. This process leads to monocyte infiltration and their differentiation into macrophages. Macrophages phagocytose lipids via scavenger receptor associated with endothelial cells I (SREC-I), resulting in the formation of foam cells and the release of key inflammatory factors, including interleukin-1β (IL-1β) and tumor necrosis factor-α (TNF-α), which amplify inflammatory signaling. The inflammatory environment promotes the phenotypic transformation of vascular smooth muscle cells (VSMCs) and activates dendritic cells (DCs). Activated DCs present antigens to T cells via major histocompatibility complex class II (MHC-II) molecules, thereby initiating adaptive immunity. T cells subsequently differentiate into various subsets, including T helper 1 cells (Th1, proinflammatory), T helper 2 cells (Th2), and regulatory T cells (Tregs, anti-inflammatory), which collectively regulate lesion progression. Tregs can transdifferentiate into T helper 17 (Th17) cells and secrete interleukin-10 (IL-10), exerting both proinflammatory and fibrotic effects. Meanwhile, B cells also differentiate into subgroups: B1 cells (B1a/B1b) produce natural immunoglobulin M (IgM), which clears ox-LDL and apoptotic cells and exerts protective effects; in contrast, B2 cells, driven by inflammatory mediators, produce pathogenic antibodies such as immunoglobulin G (IgG) and immunoglobulin E (IgE), thereby promoting disease progression. VSMCs, vascular smooth muscle cells; DCs, dendritic cells; cDCs, conventional dendritic cells; pDCs, plasmacytoid dendritic cells; TNF-α, tumor necrosis factor-α; IL-1β, interleukin-1β; IL-4, interleukin-4; IL-5, interleukin-5; IL-6, interleukin-6; IL-12, interleukin-12; IL-10, interleukin-10; ROS, reactive oxygen species; TGF-β, transforming growth factor-β; ox-LDL, oxidized low-density lipoprotein; MHC-Ⅱ, major histocompatibility complex class Ⅱ molecules; Th1, T helper 1 cells; Th2, T helper 2 cells; Th17, T helper 17 cells; Tregs, regulatory T cells; *STING*, *stimulator of interferon genes*; IFN-γ, interferon-γ; IgM, immunoglobulin M; IgG, immunoglobulin G; IgE, immunoglobulin E; ICAM-1, intercellular adhesion molecule 1 protein; MMPs, matrix metalloproteinases; NETs, neutrophil extracellular traps; SMC-Mφ, macrophage-like cells derived from smooth muscle cells; MFB, myofibroblast; PBMC, peripheral blood mononuclear cells.

The adaptive immune response in AS is characterized by a dynamic equilibrium between pathogenic lymphocytes, such as Th1 cells and follicular B2 cells, and protective lymphocytes, including Tregs and B1 cells. Subsets such as Th17 cells exhibit context-dependent duality within this framework. This understanding indicates that non-specific immunosuppression is an inherently inadequate strategy, as it may compromise essential protective functions, including those mediated by Tregs and natural IgM [73]. The future of treatment likely lies in precision immunomodulation that strategically targets harmful pathways, such as pathogenic IgG and Th1-associated cytokines, while preserving or enhancing beneficial pathways, including Treg function and IgM production. The primary challenges that remain include the limited direct translation of findings from murine models to human disease [56] and the need to identify the specific environmental cues within human plaques that influence the functional outcomes of plastic subsets such as Th17 cells and Tregs. This conceptual relationship is depicted in Fig. 1.

#### 3.2.4 Arterial Tertiary Lymphoid Organs (ATLOs)

In the early stages of AS, plaque formation is primarily driven by innate immune cells and selected adaptive immune cells, leading to gradual progression toward chronic inflammation. In advanced stages, ATLOs develop within the artery [74,75]. These structures play both a direct role in AS and serve as critical links between innate immunity, adaptive immunity, and the neurocardiovascular system [76,77]. Unlike primary and secondary lymphoid organs, which form during embryogenesis, tertiary lymphoid organs arise postnatally through the aggregation of lymphocytes and structurally resemble secondary lymphoid organs [76,78]. Under the influence of inflammatory factors and chemokines, macrophages, DCs, *CD4^+^* T lymphocytes, and Tregs migrate to the arterial adventitia [79]. While most immune cells exacerbate inflammation, Tregs exert an anti-inflammatory effect. However, a reduction in the number or function of Tregs in advanced disease can actually promote AS [80,81]. B cells also contribute to the regulation of AS through ATLOs. In *ApoE^-/-^* mice, ATLOs actively recruit both proinflammatory B2 cells and protective B1 cells to the adventitia, indicating an auto-driven immune response [81,82].

#### 3.2.5 Neuro–Immune–Cardiovascular Interfaces (NICIs)

AS has traditionally been considered primarily an immune–inflammatory disease with minimal involvement from the nervous system. However, recent research indicates that immune cells within plaques can influence nerves in the arterial adventitia, underscoring the presence of NICIs [15]. Consequently, tertiary lymphoid organs may coordinate immune responses and serve as a bridge at the NICIs, thereby affecting disease progression.

**Fig. 2.**
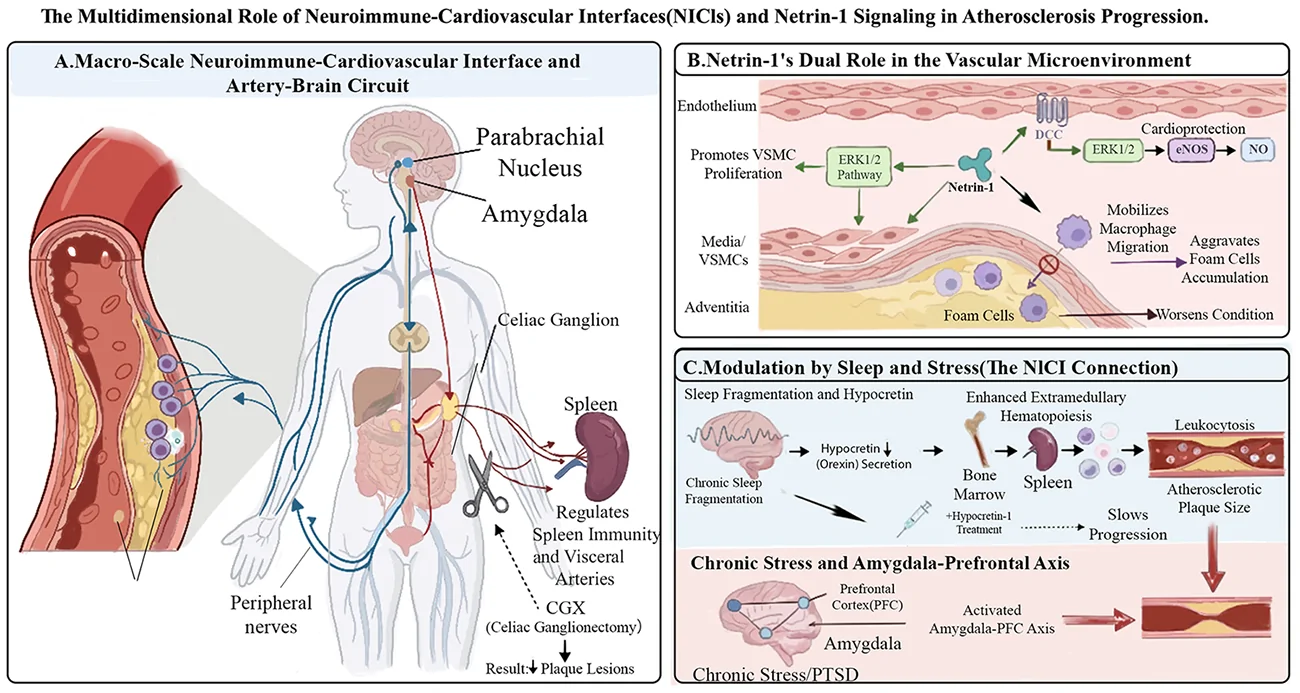
**Arterial tertiary lymphoid organs (ATLOs) establish neuro–immune–cardiovascular interfaces (NICIs) in atherosclerosis (AS)**. (A) Macroscale NICIs and the artery–brain circuit (blue and red lines). Atherosclerotic plaque-associated ATLOs establish NICIs. Afferent nerves convey signals from these sites to the brain. In response, the central nervous system regulates splenic immune function and vascular tone through efferent nerves, creating a bidirectional neuro-immune axis. Disruption of this axis (*e*.*g*., via celiac ganglionectomy, CGX) reduces lesion development. (B) Dual role of netrin-1 in the vascular microenvironment. Netrin-1 provides cardioprotective effects through the DCC/ERK1/2/eNOS pathway. Conversely, netrin-1 stimulates vascular smooth muscle cell (VSMC) proliferation and inhibits macrophage migration, thereby worsening plaque instability. (C) Modulation of NICIs by sleep and stress. Sleep fragmentation enhances extramedullary hematopoiesis and plaque progression, potentially by inhibiting hypothalamic orexin release. Chronic stress or post-traumatic stress disorder activates the amygdala–prefrontal cortex axis, directly accelerating the formation of atherosclerotic lesions. AS, atherosclerosis; CGX, celiac ganglionectomy; NICIs, neuro–immune–cardiovascular interfaces; ATLOs, arterial tertiary lymphoid organs; SLO, secondary lymphoid organs; DCC, deleted in colorectal carcinoma; ERK1/2, extracellular signal-regulated kinase 1/2; NO, nitric oxide; VSMCs, vascular smooth muscle cells; PBN, parabrachial nucleus; eNOS, endothelial nitric oxide synthase type III.

Emerging evidence suggests that NICIs play a significant role in AS [34]. For example, the neuroguidance factor netrin-1 exhibits a dual effect: netrin-1 can protect extracellular signal-regulated kinase 1/2 (ERK1/2) signaling and endothelial nitric oxide synthase type III (eNOS) activation, while also promoting disease by trapping macrophages within plaques and increasing foam cell accumulation [83]. ATLOs comprise nerves, sympathetic fibers, immune cells, and endothelial cells. Plaque formation can initiate an arterial–brain circuit that transmits signals via peripheral nerves to the spinal cord and brain. Central nuclei, including the paraventricular nucleus and amygdala, can influence immune organs via sympathetic pathways [84]. In mouse models, surgical disruption of nerve innervation, such as celiac ganglion sectioning, significantly reduces atherosclerotic plaque burden [14,85,86]. Clinical and experimental data also link poor sleep to increased cardiovascular risk. Within the NICI framework, sleep fragmentation can exacerbate plaque formation by regulating hematopoiesis. For instance, hypothalamic orexin (hypocretin-1) modulates bone marrow activity; administration of this neuropeptide significantly delayed AS progression in sleep-fragmented mice [87]. Furthermore, studies of the amygdala–frontal cortex axis show that patients with post-traumatic stress disorder (PTSD) exhibit significantly more severe atherosclerotic lesions, as indicated by brain and vascular imaging [88]. Overall, NICIs are emerging as a novel therapeutic target for AS, with the interactions among the associated components illustrated in Fig. 2.

#### 3.2.6 Immune Responses Involving Non-Immune Cells

AS involves not only immune cells but also non-immune cells, including vascular endothelial cells and VSMCs [89]. Endothelial cells are the initial participants in the immune–inflammatory response, which is triggered by underlying diseases and blood flow shear stress. Th1 lymphocytes can induce endothelial dysfunction and increase the secretion of inflammatory mediators by upregulating intercellular adhesion molecule-1 (ICAM-1) expression, thereby promoting disease progression [90,91]. The team led by Noonan et al. [92] and Wiejak et al. [93] utilized human umbilical vein endothelial cells (HUVECs), VSMCs, and THP-1 monocytes in a Transwell co-culture system to mimic the vascular microenvironment. The findings confirmed that cytokines released by HUVECs effectively recruit immune cells and stimulate the associated inflammatory responses, underscoring a fundamental aspect in early AS [92,93]. Endothelial cells also play a role in innate immunity. Li et al. [79] demonstrated that treatment of human aortic endothelial cells (HAECs) with lysophospholipids upregulates the expression of cytokines, chemokines, danger-associated molecular pattern (DAMP) receptors, and MHC-II through the activation of the nuclear factor-kappa B (NF-κB) pathway, which continuously recruits immune cells and exacerbates endothelial inflammation.

VSMCs play a crucial role in inflammation. In addition to amplifying inflammatory responses, the pronounced migratory capacity of VSMCs aids in endothelial regulation and repair [93]. Influenced by the plaque microenvironment, VSMCs can undergo phenotypic transformation, acquiring macrophage-like characteristics. VSMCs are also involved in fibrous cap formation, phagocytosis of ox-LDL, and transformation into foam cells-processes that contribute to plaque instability. Thus, VSMCs are integral to the progression of AS and are pivotal in both inflammatory and immune responses [79,91,92].

In addition to VSMCs and endothelial cells (ECs), adipocytes have attracted increasing attention [94]. Different adipose depots affect AS to varying degrees [95]. Some studies indicate that severe obesity induces hypoxia in visceral fat, activating hypoxia-inducible factor-1 alpha subunit (HIF-1α), which upregulates sphingomyelin phosphodiesterase 3 (SMPD3) and enhances ceramide production, thereby accelerating AS. Research from Wang and co-authors [96] demonstrated that reducing HIF-1α expression improved plaque conditions in *ApoE^-/-^* mice [96]. Other investigations have further suggested that endothelial injury triggers browning of perivascular adipose tissue (PVAT) and the release of neuregulin-4 (NRG4), thereby promoting M2 macrophage polarization and mitigating inflammation [97]. Epicardial fat is particularly associated with cardiovascular disease (CVDs). Adachi and co-authors [97] reported that in AS and other conditions, oxidative stress in adipose tissue increases ROS production, which aggravates local vascular inflammation and accelerates AS progression. A summary of immune cells involved in AS is presented in Table 1.

**Table 1.** **Relationship between immune cells, non-immune cells, and atherosclerosis (AS)**.

Immunity types	Cells type	Enhancement/alleviation of AS	Key proteins
Innate immunity	M1	M1 promotes AS through lipid phagocytosis and M1 polarization.	TNF-α, IL-1β, IL-6, IL-12, ROS
	M2	M2 can inhibit AS.	IL-10, TGF-β
	cDCs	The presentation of antigens to T lymphocytes through MHC-II molecules promotes AS.	MHC-Ⅱ
	pDCs	pDCs induce M1 polarization, thereby promoting AS.	IFN-γ
	Neutrophils	The release of inflammatory factors and other mediators by neutrophils can destabilize plaques and accelerate the progression of AS.	ROS, MMPs, NETs
Adaptive immunity	Th1	Th1 cells can exacerbate AS.	IFN-γ, TNF-α, IL-2
	Th2	Most studies suggest that Th2 cells play an inhibitory role in AS.	IL-4, IL-5
	Tregs	Tregs can inhibit AS.	IL-10
	Th17	Th17 cells can not only trigger inflammatory responses but also induce VSMCs to deposit collagen and stabilize plaques.	IL-17
	B1	B1 cells inhibit AS.	IgM
	B2	B2 cells have both pro‑atherogenic and anti‑atherogenic effects on AS.	IgG, IgE, IL-10
	ATLOs	Immune cells promote AS after accumulating in the arterial wall.	CXCL13, CCL21, CXCL12
	NICIs	The nervous system promotes AS by regulating the immune system.	NE, CGRP, PACAP, orexin, netrin-1
Immune responses involving non-immune cells	ECs	ECs can attract immune cells and promote AS through adhesion molecules and chemokines.	ICAM-1
	VSMCs	VSMCs promote AS.	IL-1β, IL-6, NF-κB
	Adipocytes	Hypoxia in visceral adipocytes promotes AS, whereas steatosis of adipocytes surrounding blood vessels inhibits AS.	HIF-1α, SMPD3, ceramides, NRG4

AS, atherosclerosis; ATLOs, arterial tertiary lymphoid organs; NICIs, neuro–immune–cardiovascular interfaces; ECs, endothelial cells; M1, M1 macrophages; M2, M2 macrophages; VSMCs, vascular smooth muscle cells; DCs, dendritic cells; cDCs, classical dendritic cells; pDCs, plasmacytoid dendritic cells; TNF-α, tumor necrosis factor-α; CCL2, C–C motif chemokine ligand 2; CCL21, C–C motif chemokine ligand 21; CXCL12, C–X–C motif chemokine ligand 12; CXCL13, C–X–C motif chemokine ligand 13; IL-1β, interleukin-1β; IL-2, interleukin-2; IL-4, interleukin-4; IL-5, interleukin-5; IL-6, interleukin-6; IL-10, interleukin-10; IL-12, interleukin-12; ROS, reactive oxygen species; TGF-β, transforming growth factor-β; NF-κB, nuclear factor-kappa B; Th1, T helper 1 cells; Th2, T helper 2 cells; Th17, T helper 17 cells; Tregs, regulatory T cells; MHC-II, major histocompatibility complex class II molecules; NETs, neutrophil extracellular traps; NRG4, neuregulin-4; *STING*, *stimulator of interferon genes*; IFN-γ, interferon-γ; IgM, immunoglobulin M; IgG, immunoglobulin G; IgE, immunoglobulin E; SMPD3, sphingomyelin phosphodiesterase 3; CGRP, calcitonin gene-related peptide; orexin, hypocretin-1; PACAP, pituitary adenylate cyclase-activating polypeptide; ICAM-1, intercellular adhesion molecule 1 protein; MMPs, matrix metalloproteinases; NE, norepinephrine; HIF-1α, hypoxia-inducible factor 1 alpha subunit.

## 4. Inflammation in Atherosclerosis

The primary cause of AS is activation of both innate and adaptive immunity, with LDL playing a crucial role. LDL stimulates the release of inflammatory mediators, including cytokines and chemokines such as TNF-α, which promote lesion progression. TNF-α can also disrupt the transendothelial transport of LDL [98], resulting in its accumulation within the vascular wall and further accelerating disease progression [87]. Inflammatory responses in endothelial cells recruit monocytes and macrophages to the plaque, where they engulf lipids, forming foam cells [2,13]. As foam cells release additional inflammatory factors, macrophages undergo apoptosis, leading to the formation of a necrotic core and the progression of AS to advanced stages [99,100]. Key mediators of this inflammatory response, produced primarily by innate and adaptive immune cells, include NF-κB, IL-1, IL-6, interleukin-32 (IL-32), interleukin-36 (IL-36), and interleukin-37 (IL-37) [84,101,102]. The infiltration of immune cells and the release of inflammatory factors can thin the fibrous cap of atherosclerotic plaques, thereby increasing their vulnerability. Upon plaque rupture, the necrotic core and inflammatory contents are exposed to the vascular lumen, triggering thrombosis and potentially leading to vascular stenosis or occlusion. The immune–inflammatory response in AS is highly complex and can be broadly categorized into classical and non-classical (atypical) inflammatory pathways.

### 4.1 Classical Inflammation

AS is a chronic inflammatory process that predominantly affects large- and medium-sized arteries. The hallmark features of AS include endothelial activation and immune cell recruitment. However, in contrast to typical acute inflammation, which is characterized by redness, swelling, heat, and pain, the core pathology in AS involves dysregulation of the innate immune system. This dysregulation is characterized by abnormal myeloid cell activation and excessive release of IL-1β-driven inflammatory factors, including IL-6, interleukin-18 (IL-18), TNF-α, and type I interferons. These processes are associated with activation of the NLR family pyrin domain-containing 3 (NLRP3) inflammasome and disruption of NF-κB signaling [94]. Overall, the process is highly complex, encompassing lipid accumulation, immune cell polarization, epigenetic changes, and fibrous cap formation. The inflammatory response persists at a low level and represents a non-typical, locally progressive form of vascular inflammation.

### 4.2 Atypical Inflammation

As a chronic, atypical inflammatory disease, AS primarily induces the production of inflammatory mediators, including IL-1β, IL-6, TNF-α, and hs-CRP [103]. Inflammation disseminates throughout the vessel wall but does not exhibit the typical clinical features associated with vasculitis [104,105]. Endothelial damage is the fundamental prerequisite for initiating this inflammatory process, with diabetes a significant underlying risk factor for endothelial injury [89]. In a study of 328 patients with type 2 diabetes, Stehouwer et al. [106] demonstrated that urinary albumin excretion, inflammatory markers, and endothelial dysfunction progressed concurrently and were closely associated with long-term prognosis. Meanwhile, Mazhar and colleagues [107] identified hs-CRP, a prevalent clinical marker, as being linked to conditions such as diabetes, chronic kidney disease, and autoimmune disorders. Furthermore, obesity and type 2 diabetes exacerbate insulin resistance and chronic inflammation, thereby contributing to metabolic dysfunction. Hyperglycemia results in the formation of advanced glycation end products (AGEs), mitochondrial dysfunction, and metabolic abnormalities in endothelial and myocardial cells, collectively elevating the risk of cardiovascular disease and heart failure [108]. Elevated hs-CRP levels are associated with increased rates of hospital readmission [107]. In addition to diabetes, systemic sclerosis (SSc) is frequently characterized by chronic low-grade inflammation. Heilmeier et al. [109] categorized 65 patients with SSc based on their C-reactive protein (CRP) levels and found that those with significantly elevated CRP had a higher risk of cardiovascular disease. Both CRP and lipid levels demonstrated a positive correlation with atherosclerotic disease [109]. However, some researchers argue that IL-6 is a more specific inflammatory marker than CRP after plaque rupture in AS [110]. A summary of typical and atypical inflammation is provided in Table 2.

**Table 2.** **Summary of atherosclerotic inflammatory responses**.

Feature	Classical inflammation	Atypical inflammation
Definition	Signs of acute inflammatory response: redness, swelling, heat, pain, and loss of function.	A chronic, low-grade, localized vascular inflammation without the typical features of vasculitis.
Core mechanism	Triggered by endothelial activation and immune cell recruitment.	Endothelial damage is a key trigger and is closely linked to high-risk conditions such as diabetes, obesity, and systemic sclerosis.
Key inflammatory mediators	IL-1β, IL-6, IL-18, TNF-α, NF-κB, NLRP3.	IL-1β, IL-6, TNF-α, and hs-CRP.
Pathological characteristics	Macrophages and neutrophils trigger inflammation via inflammasomes and proinflammatory factors.	AS involves lipid-driven inflammation and fibrous cap formation, where inflammation leads to AGE production and cellular dysfunction in the vessel wall.

IL-1β, interleukin-1β; IL-18, interleukin-18; TNF-α, tumor necrosis factor-α; NF-κB, nuclear factor-kappa B; NLRP3, NLR family pyrin domain containing 3; IL-6, interleukin-6; CRP, C-reactive protein; AS, atherosclerosis; AGEs, advanced glycation end products.

## 5. Impact of Anti-Inflammatory and Immunotherapeutic Strategies in Atherosclerosis

### 5.1 Anti-Inflammatory Therapeutic Strategies

Although research on inflammatory mediators in AS is ongoing, currently available anti-inflammatory treatments remain limited. Commonly used agents include statins, non-steroidal anti-inflammatory drugs (NSAIDs), and PCSK9 inhibitors [111]. Colchicine, widely used in clinical practice at low doses, can reduce NLRP3, IL-6, and hs-CRP levels, thereby attenuating plaque inflammation [112]. Nevertheless, colchicine does not significantly mitigate inflammation in acute myocardial infarction, with some studies indicating only modest benefit [113]. The primary mechanism of colchicine may involve stabilizing the fibrous cap [114]. Statins effectively lower LDL cholesterol and inflammatory markers such as NF-κB, hs-CRP, and IL-6; however, high doses are associated with increased adverse effects, thereby restricting clinical use [115].

The NLRP3/IL-1/IL-6/CRP axis is pivotal in driving chronic inflammation in AS [116]. This cascade begins with activation of the NLRP3 inflammasome, which, in turn, leads to production of IL-1β, thereby inducing IL-6 synthesis [117]. IL-6 promotes inflammatory cell infiltration and plaque instability via the JAK–STAT pathway, while also elevating CRP levels. Preclinical data substantiate the pathogenicity of this pathway, and the CANTOS trial provided significant clinical validation: targeting IL-1β interrupts this axis, reduces IL-6 and CRP levels, and yields cardiovascular benefit, thereby establishing a new paradigm for anti-inflammatory therapy in AS [116,118]. Targeting upstream inflammatory factors, such as IL-6, has shown promise; the IL-6 inhibitor ziltivekimab has been reported to improve inflammation and outcomes in AS and chronic nephritis [32]. However, most animal studies have utilized male mice, leaving potential sex-related differences in interleukin-based anti-inflammatory therapies unresolved [117]. Notably, although the CANTOS trial predominantly enrolled male patients, similar cardiovascular benefits were observed across both sexes [119].

Additionally, with the rise of the biopsychosocial medical model, it is recognized that AS may also encompass psychological issues, as mental and psychological factors contribute to its development [120]. Tricyclic antidepressants have been shown to reduce inflammation associated with AS. These medications alleviate endothelial dysfunction and plaque instability by inhibiting NF-κB activation, reducing oxidative stress, suppressing proinflammatory cytokine production, and modulating the TLR and NLRP3 inflammasome pathways [108]. Additionally, amitriptyline has been shown to reduce levels of TNF-α, ICAM-1, vascular cell adhesion molecule-1 (VCAM-1), and monocyte chemoattractant protein-1 (MCP-1) levels in HUVECs, suggesting potential cardiovascular protective effects [33].

### 5.2 Immune Therapy

In advanced AS, the accumulation of immune cells within the arterial wall leads to the formation of tertiary lymphoid organs, a process regulated by the nervous system. Within the plaque microenvironment, CD4^+^ T cells, Th1 cells, and CD8^+ ^cytotoxic T cells drive inflammation and promote plaque instability by producing IFN-γ and granzyme B. Targeting CD8^+^ T cells has shown therapeutic promise in preclinical studies [121,122]. Conversely, Tregs contribute to plaque stabilization by secreting IL-10 and transforming growth factor-β (TGF-β), making enhancement of Treg function a potential treatment strategy [121]. B cells also exert divergent effects: B1 cells are atheroprotective via the production of natural IgM antibodies, whereas B2 cells aggravate disease by generating pathogenic antibodies and activating proinflammatory T cells. Therapeutic approaches that deplete B2 cells (*e*.*g*., anti-CD20 therapy) or enhance B1 cell function have demonstrated efficacy in animal studies [121,123]. However, excessive immunosuppression may increase the risk of infection and cancer [121,122]. PCSK9 inhibitors may slow disease progression by indirectly reducing immune cell accumulation in the arterial wall, although the precise mechanisms remain unclear [82,85,124,125].

## 6. Conclusions

AS is a chronic inflammatory disease characterized by intricate interactions between innate and adaptive immunity. The pathogenesis is initiated by endothelial injury and lipid deposition, which subsequently leads to the recruitment and activation of immune cells. Proinflammatory cells, including M1 macrophages, DCs, and T cell subsets (Th1 and Th17), facilitate plaque progression, whereas Tregs and B1 cells exert protective, anti-inflammatory effects. The identification of ATLOs and NICIs underscores the complexity of immune regulation within the vascular wall.

Plaque destabilization and rupture are predominantly driven by persistent inflammation, particularly involving the NLRP3/IL-1β/IL-6 axis. Current therapies, such as statins, colchicine, and PCSK9 inhibitors, partially alleviate inflammation by lowering lipids and modulating inflammatory pathways. The CANTOS trial has confirmed the efficacy of anti-cytokine therapy as a viable treatment option. Future strategies should prioritize targeted immunomodulation of protective immune cells (Tregs and B1 cells), inhibition of proinflammatory pathways, such as IL-6 signaling, and exploration of interventions targeting neuro–immune circuits. The integration of these advancements may yield more effective plaque stabilization and a reduction in cardiovascular events.

Currently, the treatment of AS remains focused on managing the disease after diagnosis. However, as society ages, chronic inflammatory and degenerative diseases will likely necessitate targeted therapies targeting cytokines, white blood cells, or ATLOs. Indeed, such approaches are essential for stabilizing AS and reducing both mortality and disability rates.
